# Construction or updating? Event model processes during visual narrative comprehension

**DOI:** 10.3758/s13423-023-02424-w

**Published:** 2024-02-15

**Authors:** Irina R. Brich, Frank Papenmeier, Markus Huff, Martin Merkt

**Affiliations:** 1 Leibniz-Institut für Wissensmedien, Schleichstr. 6, D-72076 Tübingen, Germany; 2 Department of Psychology, Eberhard Karls Universität Tübingen, Tübingen, Germany; 3 German Institute for Adult Education – Leibniz Centre for Lifelong Learning, Bonn, Germany

**Keywords:** Narrative comprehension, Event cognition, Event models, Viewing times

## Abstract

The plot of a narrative is represented in the form of event models in working memory. Because only parts of the plot are actually presented and information is continually changing, comprehenders have to infer a good portion of a narrative and keep their mental representation updated. Research has identified two related processes (e.g., Gernsbacher, 1997): During *model construction (shifting, laying a foundation)* at large coherence breaks an event model is completely built anew. During *model updating (mapping*) at smaller omissions, however, the current event model is preserved, and only changed parts are updated through inference processes. Thus far, reliably distinguishing those two processes in visual narratives like comics was difficult. We report a study (*N* = 80) that aimed to map the differences between constructing and updating event models in visual narratives by combining measures from narrative comprehension and event cognition research and manipulating event structure. Participants watched short visual narratives designed to (not) contain event boundaries at larger coherence breaks and elicit inferences through small omissions, while we collected viewing time measures as well as event segmentation and comprehensibility data. Viewing time, segmentation, and comprehensibility data were in line with the assumption of two distinct comprehension processes. We thus found converging evidence across multiple measures for distinct model construction and updating processes in visual narratives.

## Introduction

**Fig. 1:**
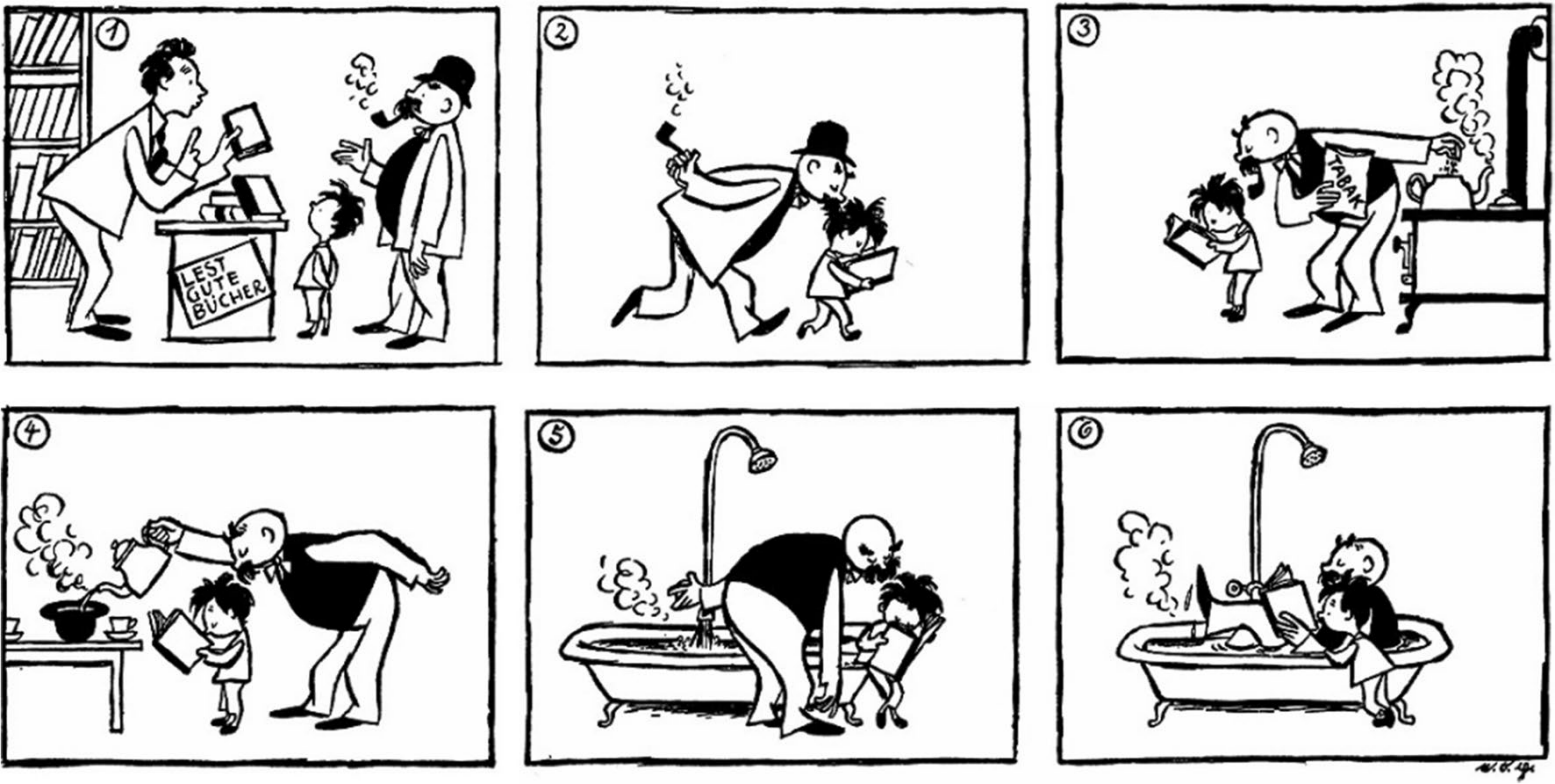
Examples of switches between two events (e.g., between Panels 1 and 2) and omissions within a single event (e.g., between Panels 5 and 6), taken from the comic strip “Das Fesselnde Buch” (“The Captivating Book”; Ohser, 2015)

Visual narratives are designed to convey a series of events in a meaningful and engaging way. To increase engagement, they employ strategies such as omissions or switching between two (parallel) events (McCloud, 1994). For example, in the visual narrative in Fig. 1, there is an omission between Panels 5 and 6, as the narrative does not explicitly depict how the father got into the bathtub. Despite this omission, Panels 5 and 6 may be considered as part of the same ongoing event because they share multiple features. In contrast, there is a more abrupt change between Panels 1 and 2, which may be interpreted as a change between two separate events. Panel 1 depicts the purchase of a book, whereas Panel 2 displays the father and the son walking. Even though the two panels are connected by the presence of the book and thus part of the same overall narrative, it can be assumed that they are depicting two separate events. Importantly, the event in Panel 1 does not conclude with the actual financial transaction, so that viewers need to infer that the book was paid for. Thus, both these techniques result in more or less severe coherence breaks that viewers need to bridge in order to make sense of the overall narrative. By systematically varying the presence of omissions within events and switches between two events, the current study tries to gain further insights into the comprehension processes occurring during these two types of narrative coherence breaks.

Narrative understanding requires comprehenders to infer a good portion of the story and to keep up with new information. Thereby, *event models*^1^ (i.e., mental representations of the story in working memory) are constructed and updated (e.g., Gernsbacher, 1997; Zacks et al., 2007). The time spent processing depicted information (i.e., reading or viewing time) represents cognitive elaboration (Radvansky & Copeland, 2010). While prolonged viewing times are related to constructing a new event model, as is the case at the beginning of a new narrative (Cohn & Wittenberg, 2015), increased viewing times also indicate elaborated information generation processes updating the current mental representation of the narrative (Huff et al., 2020; Magliano et al., 2016). The distinction between model construction and updating is important for event cognition and psycholinguistic research, as processes of attentional regulation (e.g., Huff et al., 2012) and memory formation (Huff et al., 2017) are related to these processes. Given this importance, we sought to distinguish between those two narrative comprehension processes and resolve the ambiguity in interpreting viewing times by combining them with event segmentation and comprehensibility data within visual narratives designed to contain different types of coherence breaks.

The structure-building framework (Gernsbacher, 1997; Gernsbacher et al., 1990) has shaped the fundamental understanding of the mental processing of narratives. It describes narrative understanding as an interplay of three processes, (1) *laying a foundation*, (2) *mapping* new or changing information that is largely consistent with the present structure onto it, and (3) *shifting* to a new foundation if new information is unrelated to the existing structure and mapping is too difficult. In her review article, Gernsbacher (1997) presented empirical evidence for those three processes, such as *laying a foundation* being reflected in longer reading times for the first sentence in an episode or paragraph (e.g., Glanzer et al., 1984; Haberlandt, 1980; Haberlandt et al., 1980) and the advantage of the first-mention effect (e.g., Carreiras et al., 1995; Garnham et al., 1996; Gernsbacher & Hargreaves, 1988). *Mapping* is evident in the influence of coherence cues on comprehension (faster mapping with higher coherence), such as temporal, referential, spatial, or causal coherence (e.g., Anderson et al., 1983; Black et al., 1979; Cirilo, 1981; Haviland & Clark, 1974; Keenan et al., 1984). *Shifting* is reflected in both slowed reading times (e.g., Anderson et al., 1983; Daneman & Carpenter, 1983; Mandler & Goodman, 1982) and the reduced accessibility of information from previous episodes (e.g., Gernsbacher, 1985; Haenggi et al., 1995) when structural boundaries during comprehension occur. Despite this empirical support for the structure-building framework, there is also a theoretical argument against the need for assuming its distinct processes (McNamara & Magliano, 2009). That is, whereas *mapping* is considered a central process of comprehension, McNamara and Magliano (2009) proposed that there might be no need for the assumption of *laying a foundation* and *shifting* as additional processes, because – from the viewpoint of the construction-integration model (Kintsch, 1998) – *laying a foundation* and *shifting* might be descriptive of deeper mechanisms of narrative comprehension that occur when readers encounter gaps in discourse. Thus, it remains to be resolved whether the processes proposed by the structure-building framework should be considered distinct or not. Our present work tackled this topic by investigating whether there is convergence across multiple measures regarding the distinction between event model construction (*shifting* and *laying a foundation*) and event model updating (*mapping*) in narratives with specific coherence breaks.

Model construction and updating occurs when a narrative’s plot changes on dimensions such as time, space, character, causality, and intentionality (Zwaan & Radvansky, 1998; Zwaan et al., 1995a). However, it is still debated whether observers only update the changed dimensions (incremental updating; Zwaan & Radvansky, 1998; Zwaan et al., 1995a) or whether the entire event model is reset when the plot changes (global updating; Zacks et al., 2007; Zacks, 2020). Whereas incremental updating would be compatible with *mapping* processes at plot changes, global updating would constitute an instance of *shifting* to the construction of a new model. The event indexing model (Zwaan & Radvansky, 1998; Zwaan et al., 1995a) proposes that dimension change triggers incremental updating of the event model. In contrast, event segmentation theory (Zacks et al., 2007; Zacks, 2020) assumes model updating to be global. After observers perceive an event boundary, that is a boundary between two meaningful events (Newtson, 1973; Kurby & Zacks, 2008), the entire model is reset. Such event boundaries can be identified through segmentation tasks in which participants are asked to press a button whenever they perceive that one event has ended and another has begun (Newtson, 1973; Newtson & Engquist, 1976). Kurby and Zacks (2012) proposed that participants update event models incrementally during events (e.g., the omission between Panels 5 and 6 in Fig. 1) and globally at event boundaries (e.g., the switch between two events between Panels 1 and 2 in Fig. 1), which fits with the notion of both incremental *mapping* and global *shifting* occurring during comprehension.

Recently, the scene perception and event comprehension theory SPECT (Loschky et al., 2020) integrated the processes described in the structure-building framework and research on event boundary perception. In SPECT, *shifting* and subsequently *laying a new foundation* is related to event segmentation behavior, while *mapping* is linked to inference generation processes (Graesser et al., 1994; Hutson et al., 2018; Loschky et al., 2020). The latter is needed to maintain the coherence of mental models by bridging the gaps between two or more explicitly depicted scenes or pictures (Graesser et al., 1994; Magliano et al. 2016). In contrast to low overlap between subsequent scenes, where one must *shift* to a new event (construction), bridging inferences are needed for *mapping* new information (updating) when there is a high or moderate degree of overlap indicating the continuation of an event (Hutson et al., 2018; Magliano et al. 2016).

Empirical evidence for those processes comes from discourse and text comprehension studies showing that reading times increase linearly with increasing dimension changes (Huff et al., 2018; Zacks et al., 2009), thus suggesting model updating. The presence of inference processes involved in model updating is supported with findings of longer reading times on sentences needing bridging inferences for comprehension (Haviland & Clark, 1974). Further, increased viewing times at content discontinuities between subsequent phrases can indicate event model construction (Zwaan et al., 1995b).

Recently, research showed that understanding visual narratives (i.e., comics) is based on similar processes. The viewing time of an image (i.e., comic panel) can capture cognitive processes similar to reading time in text comprehension research (Cohn & Wittenberg, 2015; Magliano et al., 2016). In particular, the viewing time paradigm is sensitive to uncovering bridging inferences (e.g., Magliano et al., 2016). Increased viewing times on the panel following an omitted or replaced one (e.g., with an action star or blank panel; Cohn & Wittenberg, 2015; Huff et al., 2020) indicate inference generation processes for the missing bridging event information. Thus, viewing time increases are supposed to be a direct measure of inference and integration processes required for *mapping* and accordingly updating an event model. However, increased viewing or reading times at the beginning of new events also indicate construction of new event models (Cohn & Wittenberg, 2015; Zwaan et al., 1995b). It is thus an open question if the viewing time paradigm used in comic research can distinguish between processes at the beginning of a new event (i.e., model construction) and the processing happening during an event (i.e., model updating).

One possible solution to resolve this ambiguity is directly manipulating the narrative event structure by introducing salient event boundaries and elicit inferences by deleting bridging event information and thus creating different coherence breaks, which require either construction or updating processes for comprehension. Another solution is combining viewing time and event segmentation measures (Magliano et al., 2012; Newtson, 1973; Kurby & Zacks, 2008). The event segmentation measure is sensitive to changes in visual narratives' semantic coherence and narrative structure (Cohn & Bender, 2017). Zacks et al. (2009) showed that sentences containing event boundaries were read more slowly. Increasing segmentation probability also led to increasing reading times related to the amount of situational change. The latter finding is consistent with evidence that segmentation magnitude increased with the number of dimensional changes (Huff et al., 2014), supporting the notion that more fundamental narrative changes, where one must *shift* to a new model, are captured in the segmentation measure. Few studies have assessed both viewing/reading times and segmentation despite recent studies showing that more than a single behavioral measure is needed to describe human event processing (Baker & Levin, 2015; Huff et al., 2018; Radvansky & Copeland, 2010).

### Experimental overview and hypotheses

This study investigates whether we can distinguish between constructing and updating a narrative's mental representation (i.e., event model) and whether viewing times are suitable for studying both processes. We report a study at the intersection of narrative comprehension and event cognition, collecting viewing times, event segmentation, and comprehension measures, while introducing event boundaries and eliciting bridging inferences to generate different types of coherence breaks in a story. As stimulus material, we used short visual narratives (e.g., Mayer, 1967). Each narrative contained a bridging event that we replaced with a blank in half of the trials (no blank and blank condition), necessitating bridging inferencing (i.e., updating). After the bridging event panel, the narrative either continued (single-event condition) or a new event began (two-events condition), inducing an event boundary (necessitating construction processes).

If *viewing times* can distinguish between updating and constructing an event model, we expect an interaction of number of events and replacing the bridging event with a blank. We expect viewing times to be generally higher for the first panel after the bridging event panel in the two-events condition, compared to the single-event condition. We also expect prolonged viewing times after the blank in the single-event condition, which indicates inference generation processes, but, as mapping is impossible at an event boundary, no effect of the blank in the two-events condition.

In addition, we expect that *event segmentation* indicates event model construction processes. Consequently, segmentation magnitude should be significantly higher on the first panel after the bridging event panel in the two-events condition (i.e., at the beginning of the second event) than in the single-event condition. In contrast, as the bridging inference processes induced through a blank are linked to updating rather than model construction processes, segmentation magnitude should not be affected by introducing blank panels.

We expect *comprehensibility* to be drastically reduced in the two events compared to the single-event condition. Because blanked information can be bridged with inferences there should only be a slight decrease in comprehensibility in the single-event condition, whereas in the two-events condition, replacing the bridging event panel with a blank should not further reduce comprehensibility due to the presence of an event boundary. We thus expect an interaction between number of events and blank.

## Methods

### Participants

Our sample consisted of 80 German university students (nine males and 51 females; 20 did not state their gender) with a mean age of 23.34 years (*SD* = 3.07; four participants did not state their age). Participants received course credit for participation. Due to an error in data collection, we excluded one participant from all analyses. For the present study (conducted in 2017) no ethics approval was required according to national and university guidelines. The conduct of the experiment followed APA standards for ethical treatment of participants.

### Apparatus and material

**Fig. 2:**
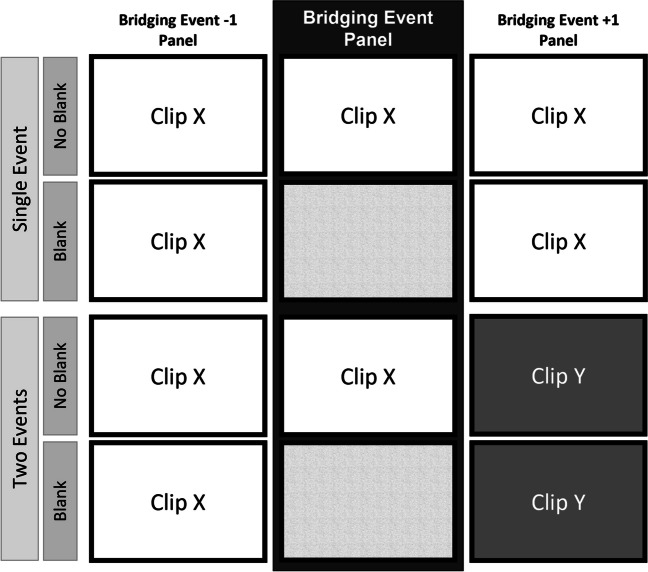
Schematic depiction of the stimulus material (three pictures surrounding the bridging event panel) in the four conditions

The basis of the stimulus material was six picture stories of the Boy Dog Frog series (Mayer, 1967, 1969, 1973, 1974; Mayer & Mayer, 1971, 1975). We used the bridging events identified by Magliano et al. (2016) as anchor points. Each of the stories contained four bridging events. In contrast to Magliano et al. (2016), we did not present the stories as a whole but separated them into 24 individual story "clips" consisting of six pictures each. We created the two-events stimuli by continuing the story with pictures/panels from another clip after the bridging event panel of the initial clip (see Fig. 2). In the two-events condition the original bridging event thus constituted the end of the previous event. Half of the trials depicted a single event, the other half two events. Further, the bridging event panel was visible in half of the trials, and for the blank condition we replaced it with a blank panel in the remaining trials (see Fig. 2). The independent variables *number of events* (single/two events) and *bridging event blanked* (no blank/blank) were manipulated within-participants. We counterbalanced the assignment of the 24 clips to the blank/no blank and single/two-events conditions across participants. The position of the bridging event was counterbalanced within participants (8x position 2, 8x position 3, 8x position 4). The 24 clips were presented in random order. Due to an error in stimulus preparation, four clips had to be excluded from all analyses, leaving seven clips with the bridging event in positions 2 and 3, and six with the bridging events in position 4.

The experiment was conducted in a laboratory setting allowing up to four participants to take part in the experiment in parallel. Both parts of the experiment (viewing time and segmentation task) were programmed with PsychoPy (Peirce, 2007, 2009) and presented on Mac-Mini with a 23-inch LCD screen.

### Procedure

The participants first provided informed consent in the lab. Participants also filled out questions for the visual fluency index (Cohn, 2014); these data are not reported here. They began the experiment with the viewing time task on the computer. The instruction stated that their task was to comprehend the depicted story and that they would see the clip on the screen one picture at a time. They could navigate to the following picture by pressing the spacebar. Instructions also stated that participants would occasionally see a gray pattern instead of a picture. After each clip, participants wrote a short summary of the depicted story to ascertain sufficient attention and involvement in comprehension (summaries were not analyzed).

After a short break, participants received the instruction for the segmentation task. All pictures of a clip were presented simultaneously in a row. Participants were instructed to select with the mouse the picture(s) that – in their opinion – constitute(s) the beginning of a new event. Selected pictures were highlighted with a transparent red overlay. Participants were allowed to mark as many event boundaries as they wanted and could also remove a mark. They confirmed their selections by pressing the space bar. After each clip, participants rated the comprehensibility of the clip on a 7-point rating scale (1: “low” to 7: “high”).

## Results

### Viewing times

We first trimmed the data using a criterion-based trimming rule considering the duration of a simple reaction and thus excluded reactions shorter than 0.48 s (141 of 9,480 trials – 1.49%) and longer than 20 s (296 of 9,480 trials – 3.12%) (Magliano et al., 2016). Second, a normative trimming was applied, excluding viewing times larger than 3 standard deviations above the arithmetical mean (after criterion-based trimming) for each experimental condition (204 trials – 2.15%).

**Fig. 3:**
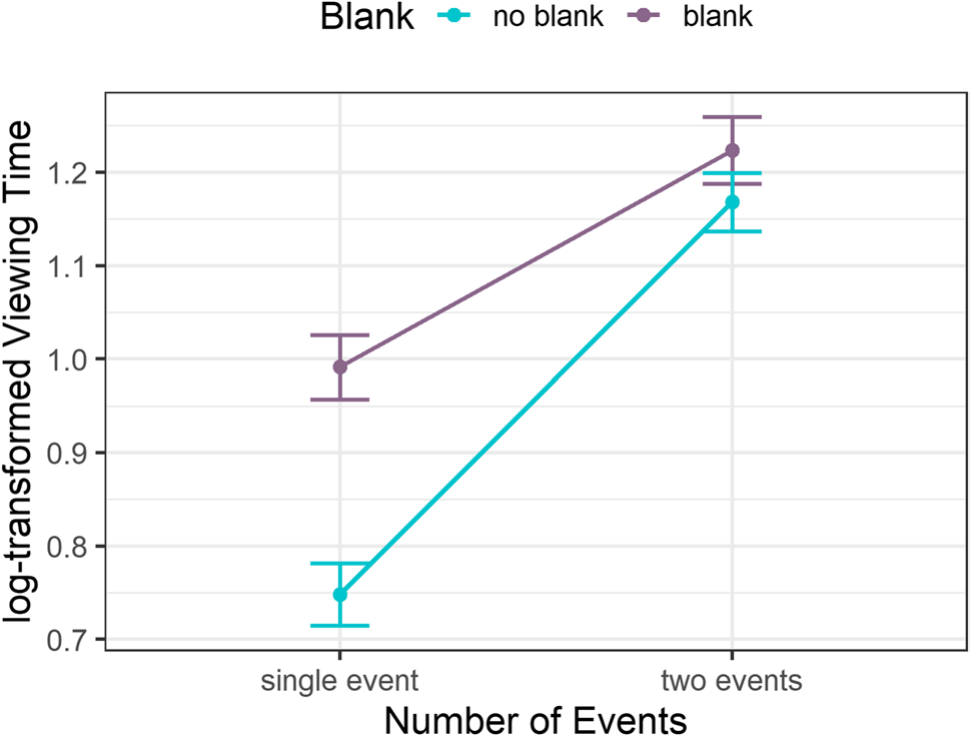
Mean log-transformed viewing time for the bridging event+1 panel dependent on number of events and blanking of the bridging event. *Note*. Error bars represent the SEM, based on the overall number of observations

We analyzed the log-transformed viewing time data for the bridging event+1 panel (Fig. 3) with linear mixed-effects models (*lme4*-package; Bates et al., 2015). The model included the *number of events* (single event, two events), *bridging event blanked* (blank, no-blank), and their interaction as fixed effects, and random intercepts for both participants and items (story clip). We analyzed the model parameters with a type-II ANOVA (*car-*package; Fox & Weisberg, 2019) and conducted additional post hoc tests with the *emmeans-*package (Lenth, 2021) with Bonferroni-adjusted p-values. Results showed effects for *number of events*, χ^2^(1) = 158.57, *p* < .001, and *bridging event blanked*, χ^2^(1) = 31.23, *p* < .001, as well as a significant interaction of the two factors, χ^2^(1) = 14.31, *p* < .001. For a single event, viewing times were higher in the blank than in the no-blank condition, *t*(1364.22) = -6.64, *p*_*adj.*_ < .001, suggesting inference processes and replicating Huff et al. (2020). In the two-events condition, however, the blank and no-blank condition did not differ, *t*(1366.02) = -1.16, *p*_*adj.*_ = .743, while viewing times were expectedly higher for two events than for single events within each blank condition (Fig. 3). Thus, our viewing time results reproduce the two event model modification processes – mapping and shifting – proposed by the structure-building framework (Gernsbacher, 1997). In particular, the blank disrupts comprehension in the single-event condition and triggers the effortful generation of bridging inferences to update the current event model (*mapping*). The overall increase in viewing times for two events compared to the single event, combined with the lack of differences between the blank and no-blank conditions for two events, indicates more elaborative processing to construct a new event model (*shifting* and *laying a foundation*). This pattern is plausible because there is no additional need to bridge the gap left by the blank when the participants build up a new model. This is further corroborated by higher processing costs at event boundaries than during bridging inferences, i.e., higher viewing times in the two-events no-blank condition than in the single-event blank condition, *t*(1366.13) = -5.07, *p*_*adj.*_ < .001.

### Segmentation

**Fig. 4:**
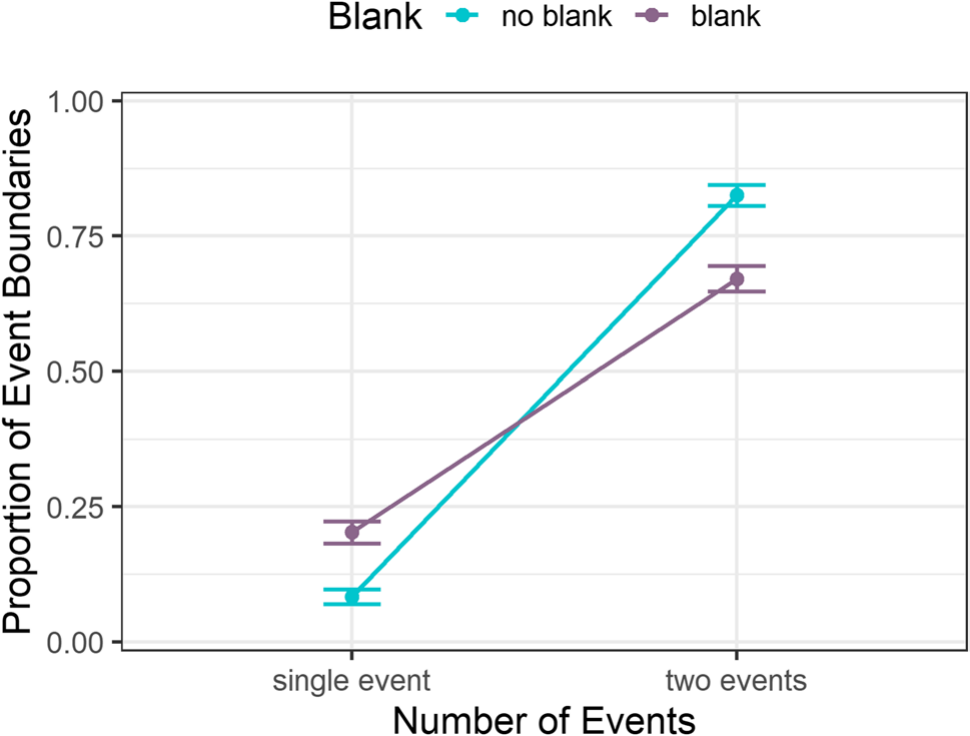
Proportion of segmented event boundaries for the bridging event+1 panel dependent on number of events and blanking of the bridging event. *Note.* Error bars represent the SEM, based on the overall number of observations

Analysis for the segmentation data at the bridging event +1 panel paralleled the viewing times except that we fitted a generalized linear mixed-effects model for the binomial segmentation variable. We observed an effect for *number of events*, χ^2^(1) = 341.59, *p* < .001, but not for *bridging event blanked*, χ^2^(1) = 1.51, *p* = .219. Further, there was a significant interaction of the two factors, χ^2^(1) = 55.60, *p* < .001 (Fig. 4). For two events, we observed less segmentation responses in the blank than in the no-blank condition at an overall higher level than the single-event condition. Thus, as expected, the beginning of a new event in the two-events condition led to a strong increase in the subjective perception of an event boundary. In the single-event condition, the relationship of blank and segmentation was reversed, i.e., higher segmentation in the blank than in the no-blank condition. Contrary to our expectations, the blank affected segmentation differently depending on the number of depicted events. The blank might have been disruptive enough for a few participants to perceive a boundary even if the same event continued in the single-event condition. Because participants saw all panels simultaneously, they probably sometimes identified the blanked picture itself as the beginning of the new event in the two-events condition, thus lowering segmentation responses on the subsequent panel. Our data for the two-events condition support this notion: while segmentations decreased on the bridging event+1 panel from the no-blank (*M* = 0.83, *SE* = 0.02) to the blank condition (*M* = 0.67, *SE* = 0.02), segmentations on the previous panel increased from the no-blank (*M* = 0.06, *SE* = 0.01, at bridging event panel) to the blank condition (*M* = 0.25, *SE* = 0.02, at blank panel).

### Comprehensibility rating

**Fig. 5:**
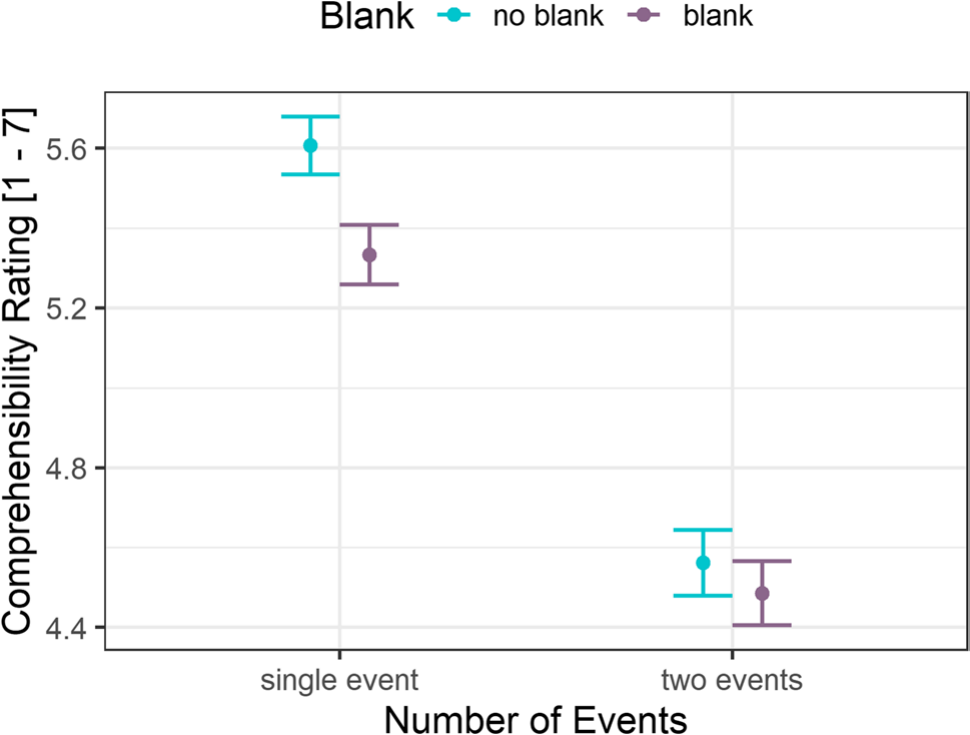
Comprehensibility ratings dependent on number of events and blanking of the bridging event. *Note.* Error bars represent the SEM, based on the overall number of observations

We analyzed the comprehensibility ratings (Fig. 5) using linear mixed-effects models (*lme4*; Bates et al., 2015) with *number of events* (single event, two events), *bridging event blanked* (no-blank, blank), and their interactions as fixed effects, and random intercepts for both participants and items. Model parameters were analyzed with a type-II ANOVA (*car*-package; Fox & Weisberg, 2019). Both replacing bridging event information with a blank, χ^2^(1) = 7.81, *p* = .005, and depicting two events in a clip, χ^2^(1) = 233.01, *p* < .001, reduced comprehensibility. There was no interaction of these two factors, χ^2^(1) = 2.54, *p* = .111.

## Discussion

To our knowledge, the reported study is the first in visual narrative research to manipulate event structure and combine measures from narrative comprehension and event cognition to identify the impact of and distinguish between the two comprehension processes, event model construction and updating. To this end, we assessed event segmentation, viewing time, and comprehensibility measures during the comprehension of short picture stories containing experimentally induced event boundaries and sections requiring bridging inferences.

With this method, we could show that viewing times reflected differences between model construction and updating processes in visual narratives. Omitting information from a visual narrative only generated additional processing effort (i.e., increased viewing times) when bridging inferences were possible during an ongoing event, thus when the current event model could be updated. However, omitting information before an event boundary did not further increase processing effort because participants had to construct a whole new event model anyway, so no further updating processes through inferences were necessary. This model construction process was captured through a large increase in viewing times at the induced event boundaries, which was above viewing time increases caused by updating processes alone. Thus, model construction after event boundaries is associated with larger cognitive costs than gaps in coherence that can be bridged during model updating. Taken together, viewing times are sensitive to model construction processes at larger changes in event structure as well as to inference processes needed for model updating at smaller omissions also for visual narratives, and provide evidence for the assumption of two different processes. Comprehensibility results further revealed that narrative understanding is largely robust against omitting information during ongoing scenes. Although there was a small decrease for blanking bridging event information, comprehensibility remained high as long as an event continued, indicating that participants were able to bridge the resulting gap through inferential mapping processes. Overall, these results provide evidence for distinct processes of *mapping* (i.e., model updating) compared to *shifting*, and *laying a foundation* (i.e., model construction) as proposed in the structure-building framework (Gernsbacher, 1997; Gernsbacher et al., 1990) and the SPECT (Loschky et al. 2020), which can be tracked through viewing times.

These findings are corroborated by the segmentation data showing that only the beginning of a new event triggered robust and marked event boundary perception. Participants clearly identified the introduced event boundaries. Blanking bridging events, however, did not trigger consistent event boundary perception processes. While the blank slightly increased event boundary perceptions during an ongoing event, segmentation was reduced for the blank condition when an induced boundary was present. Note that the latter finding was likely caused by the boundary marking procedure and thus provides no valid point for interpretation. The segmentation measure is largely sensitive to breaks in event structure and seems relatively robust against smaller omissions, and is thus suited to identify model construction processes in visual narratives.

One concern about using viewing times in comic research is whether they can capture potential differences between event model construction and updating processes. The present results using highly structured material suggest that this is possible. However, it should be noted that our experimental materials were designed as extreme examples of omissions and switches between two separate events (i.e., using blank panels and abrupt switches mid-event), which actually exist in visual narratives (see McCloud, 1994), but are not the most prototypical examples of coherence breaks in visual narratives. Therefore, the generalizability of our findings may be limited to extreme cases, whereas a distinction between mapping and shifting may not be as straightforward for less extreme cases of coherence breaks. Further, inferring cognitive processes from viewing times strongly profits from also incorporating event segmentation data. Large viewing time increases coinciding with marked segmentation could be interpreted as construction processes, while other prominent viewing time increases could be ascribed to updating. The present study underlines the importance of the perceived event structure for understanding visual narrative continuity and the collection of more than a single dependent measure.

A central contribution of this work is the converging evidence across three dependent measures (viewing times, event segmentation, comprehensibility ratings) that event model construction (*shifting* and *laying a foundation*) and event model updating (*mapping*) reflect distinct processes of narrative comprehension. This finding contradicts McNamara and Magliano’s (2009) suggestion that it is not necessary to define *shifting* and *laying a foundation* as additional processes to *mapping*. It is the interplay of our experimental design with the combination of measures of narrative comprehension (viewing times, comprehensibility ratings) and event perception (event segmentation) that has allowed us to reach this conclusion about the distinctiveness of event model construction and event model updating. Thus, comic research can not only rely on theories and findings of narrative comprehension research (e.g., Cohn, 2020; Gernsbacher, 1997), but can also profit from incorporating theories and models of event perception such as the EST (Zacks, 2020) and the Event Horizon Model (EHM; Radvansky, 2012) for further theory-building and research. Especially for other cognitive variables and consequences of model construction and updating processes, such as memory formation or attention allocation, findings from event cognition could provide a sound basis. For example, the EHM makes explicit assumptions about how event boundaries may either enhance or reduce memory depending on the characteristics of the retrieval task (Radvansky, 2012). In this context, it is argued that external cues such as walking through doorways (Pettijohn et al., 2016; Radvansky et al., 2010) or spreading information across different sections of a computer screen (Pettijohn et al., 2016) elicit the creation of new mental models and thus cause memory effects. Combining such findings of event cognition with narrative comprehension research could also be important for utilizing visual narratives in practical application scenarios, like diagnostics, mental health, or aging (e.g., Richmond et al., 2017), where event segmentation research can already make important contributions.

## Conclusion

In this study, we set out to map differences between the central comprehension processes of event model construction and updating through employing measures from narrative comprehension (i.e., viewing time) and event cognition research (i.e., segmentation) in visual narratives containing different types of coherence breaks. Results across multiple measures provide converging evidence that the assumption of a distinction between event model construction and updating has merit and should be the subject of further investigations.

## Data Availability

Data and analysis scripts (for the statistical programming language R) have been made publicly available via the Open Science Framework and can be accessed at https://osf.io/zjwe5/

## Footnotes

1 In this project, we use visual narratives to study processes of narrative comprehension. Thus, we use the term *event models* when referring to the mental representation of the depicted event. Event models are an overarching concept for situation models, which are event models derived from text, and experience models, which are event models derived from live experience (Radvansky & Zacks, 2011).

## Code availability

The code for data analysis are available in the Open Science Framework repository.
